# Comparison of supine vs head-elevated position on sensory block height following combined spinal epidural anaesthesia in Class III obese parturients undergoing elective caesarean delivery- a randomized controlled trial

**DOI:** 10.1186/s12871-026-03756-5

**Published:** 2026-03-16

**Authors:** Hayat Elfil, Aasia Afzal, Sikha S. Valappil, Gisha V. Mathew, Wafaa Y. A. Mohammed, Nesrine Belghith, Manar E. Abdel-Rahman, Tamam Alhusban

**Affiliations:** 1 Department of Anesthesia and Perioperative Medicine, Women’s Wellness and Research Centre, Doha, Qatar; 2 Department of Anesthesia, College of Medicine, Qatar University, Doha, Qatar; 3 Department of Public Health, College of Health Sciences, Qatar University, Doha, Qatar; 4 Department of Anaesthesia and Perioperative Medicine, Royal Hobart Hospital, Hobart, Australia

**Keywords:** Class III Obesity, Combined spinal epidural anaesthesia, Caesarean delivery, Head elevated laryngoscopy position, Lower segment caesarean section, Grade III obesity, Neuraxial anaesthesia, Obstetric population, Ramped position

## Abstract

**Background:**

Neuraxial anaesthesia is the preferred technique for caesarean delivery due to its superior safety profile and maternal–foetal benefits. However, general anaesthesia may still be required in emergencies or failed neuraxial attempts, particularly in obese parturients. Optimal airway management in this group is critical and the ramped position has been shown to improve preoxygenation, airway patency, and intubation conditions. While the ramped position may reduce the cephalad spread of local anaesthetic in non-obese patients, its effect in class III obese parturients remains unclear.

**Methods:**

This single-centre, prospective, randomized controlled trial was conducted between May 2023 and July 2024 at the Women’s Wellness and Research Centre, Doha, Qatar. Ninety class III obese parturients (Body mass index ≥ 40 kg/m^2^) scheduled for elective caesarean delivery under neuraxial anaesthesia were randomized to either the Standard Pillow (SP) group or the Head Elevated Laryngoscopy Position (HELP) group. All participants received combined spinal-epidural anaesthesia. The primary outcome was sensory block level, assessed 15 min after intrathecal injection using cold and pinprick tests. Secondary outcomes included block adequacy, epidural supplementation, analgesic requirements, incidence of maternal hypotension, vasopressor requirements, maternal satisfaction and neonatal outcomes.

**Results:**

Of 2,740 women screened for eligibility, 90 were randomized, with 82 included in the final analysis. HELP group: *n* = 39; SP group: *n* = 43. Baseline characteristics were comparable between groups. At 15 min post-intrathecal injection, the HELP group demonstrated a significantly lower median sensory block level (T4) compared to the SP group (T2) for both cold and pinprick sensation (*p* < 0.001). There were no significant differences between groups in terms of block inadequacy, opioid or epidural supplementation or conversion to general anaesthesia.

**Conclusion:**

Ramped positioning resulted in a lower sensory block height following spinal anaesthesia in class III obese parturients compared to standard positioning. Despite the reduced cephalad spread, block adequacy and clinical outcomes remained comparable, supporting the use of the HELP position to optimize airway access without compromising anaesthetic efficacy.

**Trial registration:**

The trial was retrospectively registered at ClinicalTrials.gov under NCT06889337 on 23rd; Feb, 2025.

## Introduction

Neuraxial anaesthesia is the preferred choice for caesarean delivery, as it avoids airway manipulation, minimizes aspiration risk, prevents foetal exposure to anaesthetics, and is associated with a reduced incidence of postpartum haemorrhage [1, 2]. However, general anaesthesia is still required in emergency cases or when neuraxial techniques fail or lead to complications. For successful intubation under direct laryngoscopy, appropriate positioning is increasingly emphasized, especially in obese patients [3, 4]. In this group of patients, the ramped position, defined as elevation of a patient’s head, shoulders and upper body in a way that the external auditory meatus is horizontally aligned with the sternal notch, has been shown to improve functional residual capacity thus prolonging safe apnoea time, [5] helps in preoxygenation more efficiently by keeping the airway patent, provides better laryngeal views, allows easier bag-mask ventilation [6] and enhances patient comfort [7]. The Troop Elevation Pillow (Troop® elevation pillow**,** Mercury Medical, Clearwater, FL, USA) helps positioning class III obese (body mass index ≥ 40 kg/m^2^), patients in a ramped position, improving airway alignment and ventilatory function for anaesthesia induction.

Ideally, all parturients—especially obese parturients—should be placed in the ramped position during caesarean delivery, regardless of the anaesthesia technique, to ensure optimal positioning for intubation if general anaesthesia is required. Two prior studies examined the effect of ramped positioning on the cephalad spread of local anaesthetic and found reduced block adequacy in the head-elevated group [8, 9]. However, neither study included class III obese parturients.

In parturients with class III obesity, physiological factors such as increased intra-abdominal pressure and reduced cerebrospinal fluid volume may influence the cephalad spread of intrathecal local anaesthetic and the distribution of neuraxial block.

We hypothesized that head elevated (ramped) position would result in a lower sensory block height compared with standard supine positioning in parturient with class III obesity undergoing caesarean delivery under neuraxial anaesthesia.

## Methods

### Study design

This was a single-centre, prospective, randomised controlled trial, which compared two parallel groups for patients. It executed from the1^st^ of May 2023 to the 11th of July 2024 at the Women’s Wellness and Research Centre (WWRC), Doha, Qatar. Ethical approval was obtained from Hamad Medical Corporation (HMC) Institutional Review Board (IRB), Protocol # MRC-01–21-032. The trial was retrospectively registered at ClinicalTrials.gov under NCT06889337.

(https://clinicaltrials.gov/study/NCT06889337?cond=NCT06889337&rank=1).

All participants provided written informed consent. A three-member DSMB reviewed the protocol before recruitment and again conducted reviews at 25%, 50%, 75%, and 100% of enrolment to monitor safety, adverse events, and intervention efficacy. The study followed CONSORT guidelines.

There was no patient and public involvement in this study planning. The research questions, outcome measures, study design, data collection, analysis, and reporting were developed and conducted solely by the research team.

### Study population

Ninety pregnant women, aged 18 years or older, classified as American Society of Anaesthesiologists (ASA) physical status III, with a BMI ≥ 40 and height between 150 and 180 cm, were included in the study. All participants had a normal singleton pregnancy, 37 weeks of gestation or beyond and were scheduled for elective caesarean delivery under neuraxial anaesthesia.

Exclusion criteria were: age < 18 years, BMI < 40, gestation < 37 weeks, preference for GA or contraindication to neuraxial anaesthesia, comorbidities other than obesity, gestational diabetes, or hypothyroidism, active labour (> 3 cm dilation with regular contractions), emergency caesarean, conditions affecting spinal anaesthesia spread (e.g., polyhydramnios, estimated foetal weight > 4 kg or oligohydramnios, suspected Intra uterine growth restriction (IUGR), and > 2 min delay between intrathecal injection and final positioning.

### Conduct of the study

All patients received oral pantoprazole 40 mg or esomeprazole 40 mg 60–90 min before surgery, as per institutional policy. On the morning of surgery, height, weight, BMI, and vertebral column (VC) length were recorded. VC length was measured from the C7 vertebra to the sacral hiatus using surface landmarks. After positioning on the operating table, abdominal girth was measured at the umbilical level at end-expiration.

Computer generated random sequence (1:1 allocation) was used to print the allocation cards which were sealed in sequentially numbered opaque envelopes by an investigator not involved in participant enrolment. The principal investigator opened the envelop in order, only when the patient arrived in the operating room after confirming eligibility and consent.

**Fig. 1:**
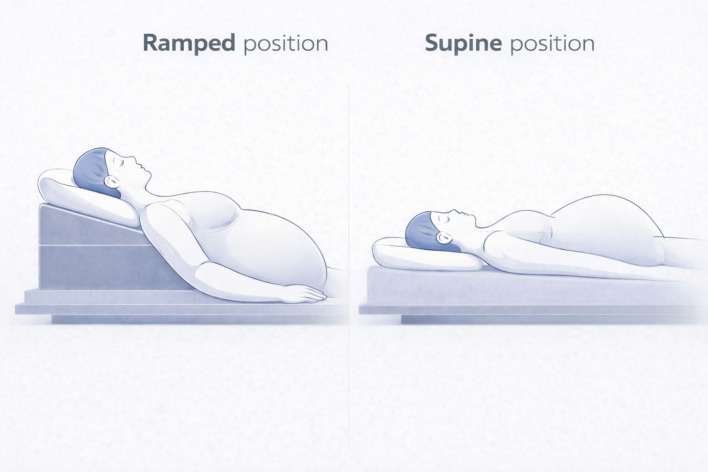
Comparison of Ramped position/Head elevated laryngoscopy position (Intervention arm) with Supine position (Control arm) in Class III obese pregnant patients

Patients in the Head Elevated Laryngoscopy Position (HELP) group were first positioned supine on a standard pillow, then on the head-elevating pillow. They were then asked to rate the comfort of the ramped position by responding to the question: “Is elevation more comfortable than lying flat?” using a 5-point Likert scale (strongly disagree to strongly agree). Standard Pillow (SP) group patients remained on the standard pillow throughout the procedure. Both the head elevated laryngoscopic position as well as the standard position have been shown in the Fig. 1.

Standard American Society of Anesthesiologists (ASA) monitoring (pulse oximetry, ECG, non-invasive blood pressure (BP), and skin temperature) was applied, and baseline values recorded. Intravenous access was secured with an 18G cannula, and Ringer’s lactate infused at 1000 ml/hour. The operating table was kept neutral, and all patients were seated for neuraxial block placement.

A single-space, needle-through-needle combined spinal–epidural (CSE) was performed at L3/L4 in the sitting position using the loss-of-resistance to saline technique. A 16G Tuohy needle identified the epidural space, followed by intrathecal injection through a 27G Whitacre needle (Smiths Medical Portex CSEcure®, Minnesota, USA). Each patient received 12.5 mg of 0.5% hyperbaric bupivacaine with 10 µg fentanyl [10, 11]. The time of intrathecal injection was recorded as “anaesthesia induction” (Time 0). The epidural catheter was advanced 4–5 cm cephalad into the epidural space. A prophylactic phenylephrine infusion (25 µg/min) was titrated to maintain systolic blood pressure within 90% of baseline. Table tilt was limited to 15° maximum, verified using the integrated inclinometer. To minimize the effects of patient positioning on intrathecal block spread, patients whose interval from induction to final positioning exceeded 2 min were excluded. The 2-min threshold was pragmatically chosen to limit potential bias from prolonged sitting while still allowing sufficient time to thread the epidural catheter and assist the patient to a comfortable supine position.

The first assessment of sensory and motor block was conducted 15 min after intrathecal administration. Sensory testing was performed by trained investigators who were not involved in patient care. Assessment was conducted bilaterally along the mid-clavicular line in a caudal-to-cephalad direction, using an evidence-based dermatome map to identify the last blocked dermatome [12]. Cold sensation was assessed using ethyl chloride spray, and pinprick sensation was evaluated with a calibrated Neurotip via the Neuropen (Owen Mumford, Oxford, UK), applying a standard force of 40 g. Motor block was assessed using the Modified Bromage Scale [13] (Table 1).

**Table 1:** Modified Bromage scale

Bromage 3	Complete motor block of lower limbs (Unable to move feet or knee)
Bromage 2	Able to move feet only
Bromage 1	Inability to raise extended leg, Able to move knee and feet
Bromage 0	No motor block

An ‘Adequate block’ was defined as *bilateral block to both sharp and cold sensation to at least the T5 dermatome, together with a modified Bromage score of 2 or 3.* The clinical decision regarding adequacy of sensory level was made on the lower of the two sensory levels (pinprick and cold).

**Fig. 2:**
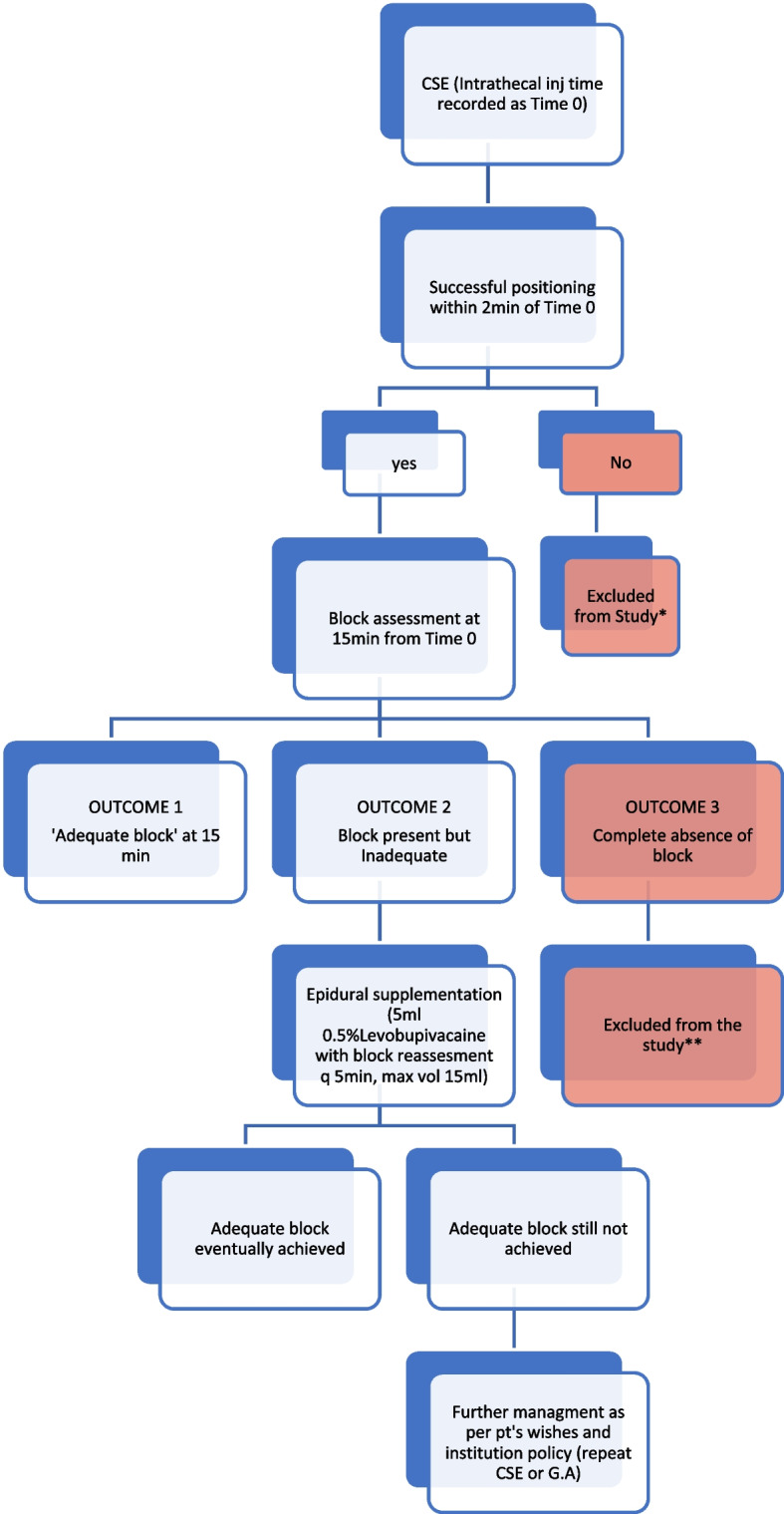
Conduct of the study with anticipated outcomes

At the initial block assessment, performed 15 min after intrathecal injection, three possible outcomes were anticipated, as outlined in the flow chart (Fig. 2). The expected outcome was the achievement of an adequate bilateral block (Outcome 1). However, there was also the possibility of an inadequate block or poor cephalad spread related to the intervention (Outcome 2). Patients in both Outcome 1 and Outcome 2 were included in the study to evaluate the effect of the intervention and head-elevated positioning on block spread.

It is also recognised that technical failure may occur with spinal or combined spinal–epidural techniques due to operator-dependent procedural or technical factors. Technical failure was defined *as absence of sensory block or absent/inadequate motor block (Bromage 0–1) after the spinal component of CSE, assessed at 15 min post spinal injection.* As the effect of any intervention on block spread cannot be studied when the block is not present, these patients were excluded from the study (Outcome 3).

Once an adequate block was confirmed—either following the initial intra-thecal injection or after subsequent epidural supplementation—surgery was commenced. If a patient subsequently experienced intraoperative discomfort or pain, further management was not protocol-driven; the dose/volume of intravenous or epidural supplementation was carefully documented but left to the discretion of the primary anaesthetist.

Intra operative hypotension was defined as systolic BP ≤ 90% of baseline. A prophylactic phenylephrine infusion (25 µg/min) was initiated for all patients and titrated as needed, with additional phenylephrine or ephedrine boluses administered per institutional protocol to maintain target BP.

After surgery, if the patient belonged to the head elevation group, the primary surgeon was asked: ‘If he/she thought the elevated position of the patient negatively impacted the ease of performing surgery i.e. made it more difficult to perform’. The responses were similarly recorded using a 5-point Likert scale.

### Outcomes

The primary outcome was the dermatomal sensory level, defined as the loss of cold and sharp sensation, measured 15 min after the intrathecal administration.

Secondary outcomes included inadequate sensory block height at 15 min; the need for intravenous opioids and epidural supplementation; conversion to general anaesthesia; incidence of maternal hypotension (defined as systolic blood pressure ≤ 90% of baseline) and vasopressor requirements. Maternal satisfaction with the ramped position; and neonatal outcomes (birth weight, Apgar scores, umbilical arterial and venous pH, standardized base excess, and lactate levels) were also recorded as secondary outcomes.

### Adverse events(AE)/Severe Adverse events (SAE)

Adverse events were defined as unintended maternal or neonatal events that resulted in harm or required care beyond standard care. Serious adverse events included life-threatening illness/injury, permanent or significant disability, need for intervention to prevent permanent impairment, foetal distress or death, or maternal death. Severity, causality, and actions taken were documented for all events.

Inadequate block height and intraoperative pain were prespecified secondary outcomes used to assess the adequacy of the primary anaesthetic technique and intervention. Episodes of inadequate block height or breakthrough pain were anticipated and managed according to the institutional protocol with supplemental analgesia and/or epidural supplementation. These events were not classified as adverse events unless they were unexpected, resulted in harm, or required deviation from standard management. Conversion to general anaesthesia was prespecified as a secondary outcome reflecting anaesthetic technique inadequacy. Given the increased maternal and foetal risks associated with general anaesthesia in pregnant patients and the availability of epidural backup, conversion to general anaesthesia was also classified as an adverse event and reported accordingly.

In accordance with CONSORT harms guidance, harms were assessed using both systematic and non-systematic approaches. Systematic assessment included predefined sensory and motor block assessment, structured documentation of secondary outcomes including need for iv analgesia or epidural supplementation, vasopressor use, conversion to general anaesthesia and neonatal outcomes. Non-systematic assessment included spontaneous reporting of adverse events or safety concerns by clinical staff or research team members during routine care.

### Statistical analysis

To detect a minimum clinically important difference of 2.0 (SD 3.0) dermatomes between the groups with 80% power and *P* < 0.05, a minimum of 37 subjects per group were required [14] . The sample size was increased by 20%, resulting in *n* = 45 per group, to account for non-parametric adjustments and potential drop-outs.

The primary outcome was the dermatomal height of sensory block in both groups. Dermatomes from C2 to S5 were numbered from 1 to 29 and treated as ordinal data. Continuous variables were summarized as mean (SD) for normally distributed data and median (IQR) for skewed distributions, with comparisons made using Student’s t-test and the Mann–Whitney U-test, respectively. Distribution of continuous variables was assessed using descriptive statistics, graphical inspection using histograms, and Shapiro–Wilk test for normality. Categorical variables were expressed as frequencies and percentages, with comparisons performed using the Chi-square or Fisher exact tests. Boxplots were used to visually represent sensory block levels (loss of cold and pinprick sensation) at 15 min.

In accordance with Institutional Review Board (IRB) requirements, the original study protocol specified three interim analyses and one final analysis to be conducted at 25%, 50%, 75%, and 100% participant enrolment, with predefined criteria for early trial stopping for safety. Following the first interim analysis at 25% enrolment, no safety concerns were identified, and the IRB approved continuation of the trial with a single final analysis. The study protocol was subsequently amended to reflect this modification.

All analyses were conducted using Stata version 18.5 (StataCorp, College Station, TX, USA) [15], with statistical significance set at *p* < 0.05.

## Results

**Fig. 3:**
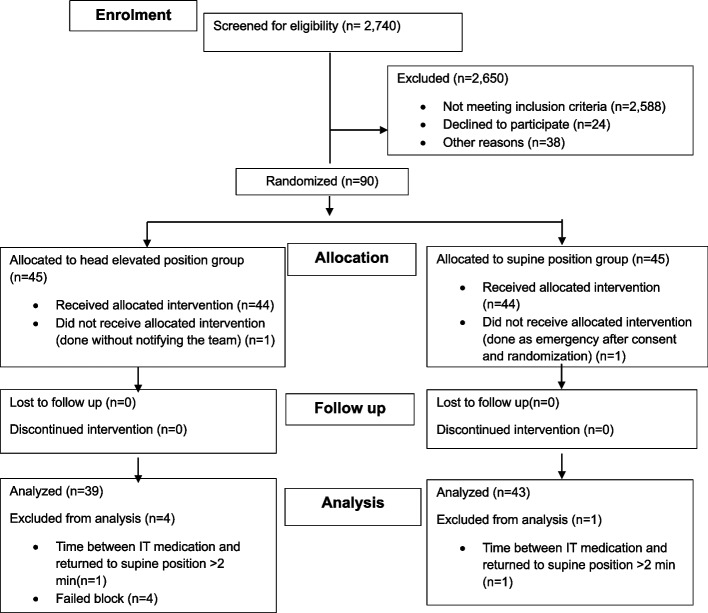
Consort Flow diagram for patient enrolment and allocation

The CONSORT flow diagram (Fig. 3) summarizes patient enrolment. Electronic health records of 2,740 women scheduled for elective lower segment caesarean section (LSCS) during the study period were screened to identify eligible participants. Of these, 2,588 did not meet the inclusion criteria. Among the remaining women, 24 declined participation, 20 underwent emergency caesarean delivery, and 18 were further excluded (2 requested general anaesthesia, 5 had restricted access due to privacy or security constraints, and 11 due to research team unavailability).

Ninety patients were therefore consented and randomized: 45 to the Standard Pillow (SP) group and 45 to the Head-Elevated Laryngoscopy Position (HELP) group. One patient in each group did not receive the assigned intervention due to lack of research team notification or emergency caesarean delivery. Additionally, five patients in HELP group were excluded (four for neuraxial technique failure and one for delayed positioning > 2 min). One patient in the SP group was excluded for the same timing non-compliance. Ultimately, data from 39 patients in the HELP group and 43 patients in the SP group was included in the final analysis for all primary and secondary outcomes. Baseline maternal demographics, haemodynamic parameters, and operative characteristics were comparable between the two groups, with no statistically significant differences observed (Table 2).

**Table 2:** Maternal demographics, baseline hemodynamic parameters and operative data

	**Standard Pillow** **Group** **(*n* = 43)**	**Elevation Pillow** **Group** **(*n* = 39)**	*p*-value^*^
Age (years), Median [IQR]	36.0 [7.0]	34.0 [6.0]	0.156
Gravida, Median [IQR]	4.0 [3.0]	4.0 [2.0]	0.386
Parity, Median [IQR]	2.0 [2.0]	2.0 [1.0]	0.865
Previous CS, Median [IQR]	2.0 [2.0]	2.0 [1.0]	0.430
Weight (kg), Mean (SD)	110.5 (10.2)	111.1 (11.8)	0.802
Height (cm), Mean (SD)	159.2 (5.2)	158.6 (5.0)	0.610
BMI, Mean (SD)	43.6 (3.2)	44.1 (3.9)	0.515
Vertebral column length (cm), Mean (SD)	53.2 (3.7)	53.3 (3.8)	0.941
Abdominal girth (cm), Mean (SD)	125.1 (8.0)	125.1 (10.5)	0.996
Temperature (C^o^), Mean (SD)	36.9 (0.4)	37.0 (0.4)	0.262
Heart rate (bpm), Mean (SD)	92.7 (12.9)	91.1 (16.3)	0.618
SPO2 (%), Mean (SD)	99.0 (1.0)	98.8 (0.9)	0.536
SBP (mmHg), Mean (SD)	125.8 (10.7)	122.3 (10.0)	0.125
DBP (mmHg), Mean (SD)	77.7 (11.5)	78.5 (10.3)	0.745
Duration in minutes, Median [IQR]			
Skin incision to delivery	8.0 [6.0]	8.0 [4.0]	0.913
Skin incision to uterine incision	7.0 [4.0]	7.0 [4.5]	0.704
Uterine incision to delivery	1.0 [1.8]	1.0 [0.3]	0.185
Skin incision to end of surgery	45.0 [21.0]	51.0 [16.0]	0.318
Time 0 to delivery	29.0 [5.0]	28.0 [6.0]	0.448

^*^ Student test when reporting mean (SD), Mann–Whitney U- test when reporting median [IQR], Chi-square test when reporting percentages

### Primary outcome

**Fig. 4:**
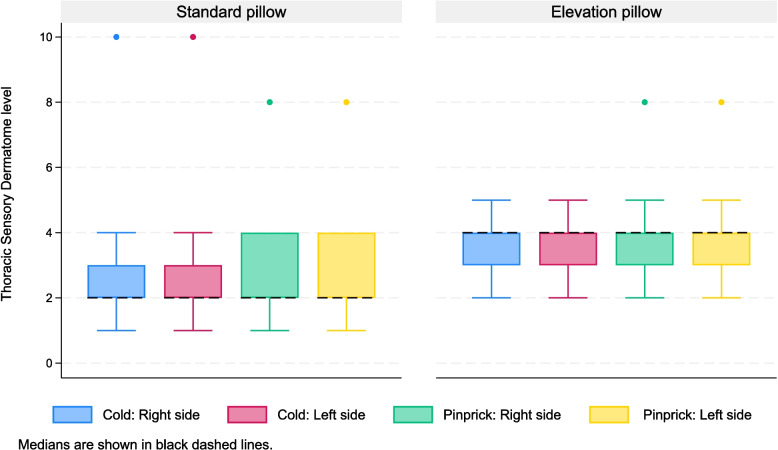
Boxplots illustrating bilateral sensory levels to cold and pinprick at 15 min in both the standard pillow and elevation pillow groups. Data presented as Median (Black dashed line), IQR (box), whiskers (1.5 × IQR), coloured dots (outliers)

At 15 min post intrathecal injection, the median dermatomal level for both cold and pinprick was T2 in the SP group and T4 in the HELP group—a two-dermatome difference that was statistically significant (*p* < 0.001, Student’s t-test). There were no differences in block height between left and right sides for any sensory modality in any individual. (Fig. 4).

### Secondary outcome

There were no significant differences between groups in block inadequacy at 15 min, achievement of adequate block after epidural supplementation, need for epidural or opioid supplementation, or conversion to general anaesthesia (GA).

Inadequate block height at 15 min was observed in 2 patients, one in each group—both of the patients achieved adequate block with epidural supplementation. Similarly incidence of intraoperative pain after establishing an adequate block–measured indirectly by the analgesic requirements–.was similar in both groups (two patients in the control group vs. three in the intervention group required additional analgesia). Conversion to general anaesthesia occurred in 1 patient in the head-elevated group and none in the standard pillow group (Table 3).

**Table 3:** Secondary outcomes

	**Standard Pillow** **Group** **(n = 43)**	**Elevation Pillow** **Group** **(n = 39)**		*p*-value*
**Secondary outcomes **				
**Initial block ‘inadequate’:**				
Inadequate block height at 15 min (n)	1	1	1.000	
Adequate block after epidural supplementation (n)	1	1	1.000	
**Initial Block ‘adequate’:**				
Intraoperative iv fentanyl supplementation (n)	2	2	1.000	
Intraoperative epidural supplementation (n)	1	3	0.348	
Conversion to general anaesthesia (n)	0	1	0.476	

^*^Student t-test when reporting mean (SD), Mann–Whitney U- test when reporting median [IQR], Chi-square test when reporting percentages

The incidence of maternal hypotension was numerically lower in the head-elevated position group compared with the standard supine group (25.6% vs. 44.2%, respectively); however this difference did not reach statistical significance (Pearson χ^2^ = 3.08, p = 0.079).The total phenylephrine consumption was similar between the groups, though the mean ephedrine dose among patients who received it was significantly higher in the SP group compared to the HELP group (11.0 ± 3.1 mg vs. 6.0 ± 0.0 mg; p = 0.031, Student’s t-test).

There were no significant differences in neonatal outcome between the two groups, Although the difference in umbilical venous pH between the groups reached statistical significance (p = 0.017), all measured values were within normal clinical range and therefore not clinically significant. (Table 4).

**Table 4:** Neonatal outcomes

	**Standard Pillow** **Group** **(*n* = 43)**	**Elevation Pillow** **Group** **(*n* = 39)**	*p*-value^*^
**Neonatal outcomes:**			
Infant weight (g), Mean (SD)	3403.605 (359.328)	3366.897 (405.904)	0.665
APGAR score at 1 min, Median [IQR]	9.0 [0.0]	9.0 [0.0]	0.341
APGAR score at 5 min, Median [IQR]	10[0.0]	10[0.0]	1.000
Umbilical Artery pH, Mean (SD)	7.299 (0.040)	7.312 (0.038)	0.126
Umbilical Artery Lactate, Median [IQR]	1.600 [0.800]	1.600 [0.700]	0.945
Umbilical Artery Base Excess, Mean (SD)	-0.355 (2.257)	-0.013 (1.607)	0.442
Umbilical Venous pH, Median [IQR]	7.343 [0.046]	7.365[0.032]	0.017
Umbilical Venous Lactate, Median [IQR]	1.400 [0.500]	1.400 [0.400]	0.802
Umbilical Venous Base Excess, Median [IQR]	-1.050 [2.500]	-0.750 [1.400]	0.541

^*^ Student t-test when reporting mean (SD), Mann–Whitney U- test when reporting median [IQR], Chi-square test when reporting percentages

**Fig. 5:**
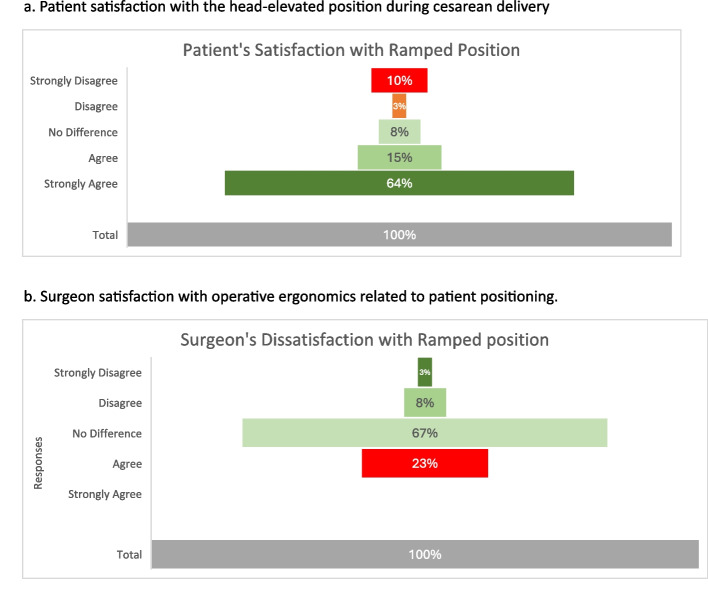
**a** Patient satisfaction with the head-elevated position during cesarean delivery. **b** Surgeon satisfaction with operative ergonomics related to patient positioning

Maternal responses on a 5-point Likert scale (strongly disagree to strongly agree) to indicate if elevation was more comfortable than lying flat?” Similarly, Surgeons documented their response to indicate if the ramped position negatively impacted their ability to perform the surgery (Fig. 5).

### Adverse events

As conversion to general anaesthesia was predefined as both a secondary outcome and an adverse event, one such adverse event was reported to the IRB in the intervention group.

## Discussion

To our knowledge, this is the first prospective randomized controlled trial to examine the effect of the ramped position on neuraxial block height in parturients with Class III obesity undergoing caesarean delivery with CSE anaesthesia. Our findings demonstrate that the sensory block height required for successful caesarean delivery can be reliably achieved in the head-elevated position, without increasing the need for epidural or intravenous supplemental analgesia.

Previous studies assessing the head-elevated position during caesarean delivery under neuraxial anaesthesia reported an increased need for epidural supplementation (Cheesman et al., 2014) [8] and a lower sensory block level, along with higher rates of epidural supplementation and conversion to general anaesthesia (Elfil et al., 2016) [9]. These findings presented a dilemma for anaesthetists: although the ramped position improves respiratory reserve, maintains airway patency, and enhances laryngoscopic views in obstetric patients, it was thought to increase the risk of neuraxial technique failure. However, the mean BMI in these earlier studies was 29 and 26, respectively, limiting the generalisability of their conclusions to women with Class III obesity.

Our study not only confirmed a reduced cephalad spread of neuraxial block in the head-elevated position (median sensory block of T2 in the control group vs. T4 in the intervention group) but also provided reassuring evidence that, in Class III obese parturients—the subgroup most likely to benefit from the ramped position—adequate block height can still be reliably achieved.

A plausible explanation for the observed differences lies in the anatomical and physiological variations between pregnant patients with normal and elevated BMI, particularly the link between higher BMI and reduced cerebrospinal fluid (CSF) volume, which leads to a greater cephalad spread of local anaesthetic [16]. CSF volume can be reduced by several mechanisms that apply external pressure to the dural sac. These include increased intra-abdominal pressure caused by excess abdominal fat; engorgement of the epidural venous plexus due to compression of the inferior vena cava, and consequent redirection of venous return; and inward displacement of soft tissues—especially adipose tissue—within the intervertebral foramina, all contributing to CSF displacement [16, 17]. In Class III obese parturients, this reduction in CSF volume likely facilitated achieving the desired block height even when ramped.

Our findings highlight an additional key consideration: the importance of selecting an appropriate spinal anaesthetic dose. Cheesman et al. and Elfil et al. used 11.25 mg and 11 mg of hyperbaric bupivacaine, respectively—both notably lower than the 12.5 mg dose used in our study. The considerable variation in the literature regarding the optimal spinal dose for caesarean delivery suggests that a single standard dose may not be suitable for all patients. The spread of local anaesthetic is influenced by multiple factors, including various patient-related factors (weight, vertebral column length, abdominal girth), drug-related factors (dose, baricity) and technique-related factors (patient position during injection, injection level, injection speed) [18–20] Ginosar et al. (2005) [21] conducted a prospective, randomized, double-blind dose-finding study in non-obese parturients undergoing elective caesarean delivery and using a logistic regression model, reported an ED95 of 11.2 mg hyperbaric bupivacaine (co-administered with opioids) for successful surgery. Using the same methodology at the same institution, Carvalho et al. (2011) [11] studied parturients with a BMI ≥ 40 and reported an ED50 of 9.8 mg, with a mathematically extrapolated ED95 of 15 mg; however, the highest administered dose in that study was 11 mg. Our study was conducted at a large tertiary centre performing approximately 18,000 deliveries per year. The standard intrathecal dose for most caesarean deliveries under spinal anaesthesia is 12.5 mg ± 0.5 mg of hyperbaric bupivacaine with fentanyl at our institution. For this study, we used the same 12.5 mg dose in patients with BMI ≥ 40 to compensate for the potentially reduced cephalad spread associated with a head-elevated position. However, if the head-elevated position is not utilized, this dose should be used with caution in high-BMI patients, as evidenced by the higher (T2) block levels observed in our control group.

We chose a 2-min maximum sitting period after intrathecal injection to avoid bias from prolonged sitting affecting block height. Previous clinical studies have shown that varying durations in the sitting position influence the cephalad spread of hyperbaric bupivacaine. In this study (Ariyama et al., 2009), [22] patients remaining seated for only 2 min after injection achieved significantly higher sensory block levels than those kept sitting for 5 min or longer (approximately T5 vs T9 and T11, respectively), indicating that longer sitting limits cephalad spread.

Inadequate block height at 15 min after spinal injection, was observed in only two patients—one in the control group and one in the intervention group—a difference that was not statistically significant. In both the cases, the block was successfully supplemented with an epidural top-up using 0.5% levobupivacaine. Intraoperative pain, after establishing an adequate block, was assessed indirectly by the requirement for supplemental analgesia. There was no significant difference between groups (two patients in the control group vs. three in the intervention group required additional analgesia). Pain management followed departmental protocols, including reassurance, intravenous fentanyl, epidural supplementation, or conversion to general anaesthesia. In the control group, both patients received intravenous fentanyl, with one also requiring epidural supplementation. In the intervention group, all three patients received epidural supplementation, two were given intravenous fentanyl, and one required conversion to GA. Consistent with this, conversion to GA did not differ significantly between groups (none vs. one). The only patient requiring GA had a history of chronic neuropathic pain and possibly altered pain perception. The presence of an epidural catheter in our study population may have lowered the anaesthesiologist’s threshold for administering supplemental epidural anaesthesia, compared with a single-shot spinal technique, where options are limited to systemic analgesia or GA conversion.

To prevent spinal hypotension, a phenylephrine infusion was commenced at 25 mcg/min and titrated to maintain systolic blood pressure within 90% of the baseline value. Total phenylephrine consumption did not differ significantly between the groups. However, ephedrine use was significantly higher in the control group. This could be attributed to the higher sensory block level observed in this group, potentially inhibiting cardio-acceleratory fibres and subsequent bradycardia, prompting anaesthesiologists to prefer the use of ephedrine over phenylephrine. The head-elevated position proved safe in our study population and did not delay foetal delivery. All surgical time intervals—including skin incision to uterine incision, uterine incision to delivery, and skin incision to the end of surgery—were comparable between groups. Neonatal outcomes were also similar. However, the study was not powered to detect statistically significant differences in secondary outcomes. Parturients found the ramped position significantly more comfortable. Blinding the operating surgeons to patient positioning was not feasible. While majority of the surgeons reported no noticeable difference or felt that the ramped position did not adversely affect the ease of performing surgery, 23% (*n* = 9) expressed a preference for the standard position.

The major strength of this work is its distinct contribution as the first randomized controlled trial to assess the influence of the ramped position on neuraxial block height in obese parturients. Additionally, the randomized, prospective design helped us minimize selection bias and confounding, ensuring comparable baseline characteristics between groups. The use of objective, quantifiable outcomes—such as dermatomal sensory block height measured with a calibrated Neuropen while referencing the evidence based dermatomal map—enhanced measurement precision and reproducibility. Oversight by an independent Data Safety Monitoring Board and adherence to CONSORT reporting guidelines ensured ethical integrity and transparency. One limitation of the study is that we did not use loss of touch to assess the block height, which is considered clinical and medicolegal standard for evaluating the adequacy of the spinal anaesthesia for caesarean delivery [23]. As a loss of cold sensation is the standard method used in our institution, we were concerned that unfamiliarity with alternative testing modalities could potentially introduce error. To enhance the reliability of our assessments, we opted to employ a combination of two modalities (pinprick and cold). Sensory block assessment was not limited to a single investigator, and the involvement of multiple assessors may have introduced inter-observer variability. Although standardised assessment tools (Neuropen and evidence-based dermatome map) were used, some degree of measurement variation cannot be excluded. Additionally, standardized pain scores were not used to assess intraoperative pain and its severity, as pain was evaluated indirectly through the need for supplemental analgesia. Blinding of investigators and participants to the intervention was not feasible, representing another key limitation of the study.

## Conclusion

In summary, our study demonstrates that an adequate sensory block necessary to successfully perform caesarean delivery under CSE anaesthesia in parturients with Class III obesity can be reliably achieved in a head-elevated position, without increasing the need for intravenous or epidural supplemental analgesia. However, as the study was conducted using a CSE technique, the generalizability of these findings to settings employing a single-shot spinal anaesthesia in the head elevated ramped position remains uncertain. Future studies with large sample size to validate the findings are required. Additionally, a dose finding study in this specific cohort of patients in the presence of ramped position could yield informative result.

## Data Availability

The study protocol, the data sets used and analysed, and the statistical analysis plan for the current study are available from the corresponding author on reasonable request.

## Abbreviations

ASA: American Society of Anaesthesiologists
BMI: Body Mass Index
LSCS: Caesarean delivery (CD) or lower segment caesarean section
CSF: Cerebrospinal fluid
CSE: Combined spinal-epidural anaesthesia
GA: General anaesthesia
HMC: Hamad Medical Corporation
HELP: Head Elevated Laryngoscopy Position
IRB: Institutional Review Board
IUGR: Intra uterine growth restriction
SP: Standard Pillow
T: Thoracic
VC: Vertebral column
